# Effects of a Faith-Based, Multilevel Intervention on HIV-Related Stigma and HIV Knowledge Among African American Church-Affiliated Populations

**DOI:** 10.1007/s40615-025-02436-3

**Published:** 2025-04-25

**Authors:** Kathryn P. Derose, Jannette Berkley-Patton, Carole Bowe Thompson, Tacia Burgin, Chavon Hamilton-Burgess, Eric Williams, Cassandra Wainright, Stephen Simon, Jenifer E. Allsworth

**Affiliations:** 1 Department of Health Promotion & Policy, University of Massachusetts Amherst, 715 N. Pleasant St, Amherst, MA 01003 USA; 2 Department of Behavioral and Policy Sciences, RAND, 1776 Main Street, Santa Monica, CA USA; 3 University of Missouri–Kansas City, School of Medicine, 2411 Holmes, Kansas City, MO 64108 USA; 4 Calvary Community Outreach Network, 3002 Holmes, Kansas City, MO 64109 USA; 5 Heaven Sent Outreach Ministries, 11200 E 75 Th Terr, Raytown, MO 64138 USA

**Keywords:** HIV stigma, Faith-based organizations, African American populations, Interventions

## Abstract

**Background:**

HIV-related stigmas contribute to HIV disparities, which faith-based organizations could address, but few studies have measured faith-based HIV intervention effects on HIV-related stigmas.

**Methods:**

Taking It to the Pews (TIPS) is a multilevel, religiously tailored HIV prevention intervention developed and implemented with faith leaders. Over 12 months, trained church health liaisons implemented the TIPS Toolkit (e.g., HIV sermon guides and responsive readings, testimonials) with the primary aim of increasing HIV testing. A cluster randomized controlled trial with 14 predominantly African American churches in Kansas City, MO, compared TIPS to a non-tailored, multilevel HIV education intervention; both intervention and comparison groups offered church-based HIV testing events with the local health department. We examined whether TIPS affected HIV stigma among congregants and community members, specifically, HIV discomfort (5-item scale), anticipated HIV stigma (4-item scale), and overall HIV stigma (11-item scale), while controlling for known correlates of HIV stigma.

**Results:**

We recruited 1491 church and community members at 14 churches. Compared to standard HIV education, TIPS did not decrease HIV discomfort or overall HIV stigma and did increase anticipated HIV stigma. A secondary analysis found that among intervention participants, intervention exposure was associated lower stigma and higher HIV knowledge, with HIV testing events and information from health professionals or HIV + people being particularly influential.

**Conclusion:**

Direct contact with health professionals and HIV + people can help reduce stigma among church-affiliated populations, but broader exposure and strategies are needed for congregation-level stigma. Stigma reduction and HIV testing may have synergistic effects in faith-based settings.

**Supplementary Information:**

The online version contains supplementary material available at 10.1007/s40615-025-02436-3.

## Introduction

In 2022, Black and African American persons comprised 13% of the US population but 38% of US HIV diagnoses. These HIV disparities experienced by African American populations in the USA are perpetuated by societal and structural factors, including limited access to health care and HIV testing [1–3]. Since early HIV treatment is still the most effective way to prevent transmission of HIV [4, 5], improving access to HIV testing for African American populations is paramount for their own health and well-being and population health overall. Community-based, accessible HIV testing has been shown to increase HIV testing among minoritized and underserved populations [6], as it helps overcome structural barriers, such as the lack of access to transportation and/or the lack of trust in the medical system due to previous experiences of healthcare discrimination [7].

In addition to increasing access to HIV testing, reducing the stigma associated with HIV is needed to ensure that people feel comfortable getting tested and seeking treatment. Previous research has conceptualized different types of stigma that are important in shaping people’s attitudes towards HIV: *instrumental* stigma refers to concerns about the potential consequences of interacting with a stigmatized person, such as becoming infected with HIV, while *symbolic* stigma refers to concerns about what the stigmatized condition, such as HIV, symbolizes [8, 9]. Instrumental stigma can translate into feelings of discomfort about interacting with HIV + individuals, while symbolic stigma affects how people think they might be treated by others if they had HIV (anticipated stigma or rejection).

The USA has identified faith-based organizations, such as African American churches, as integral partners in expanding access to HIV testing and care, as they are trusted sources of information and strategically located in communities that are most impacted [10]. African American faith-based organizations have historically played a pivotal role in social justice movements in the Black community [11], and many have ongoing health ministries with their congregations and communities [12, 13]. Systematic reviews of faith-based interventions across a range of health topics have found most have been done in and with predominantly African American congregations [14–16]).

Although there have been numerous reports in the literature about congregation-based HIV prevention and testing interventions [17], few have examined the extent to which these HIV prevention and testing interventions have affected HIV stigma. HIV testing and HIV-related stigma reduction have been described as mutually facilitative in congregational settings [18], as they “normalize” HIV as a health issue rather than a moral issue [19, 20]. Stigma reduction within churches may contribute to congregants’ willingness to get tested in church settings. Also, church-based HIV testing may reinforce the perception that the HIV epidemic is present in the community served by the congregation, making it harder to see HIV as only affecting “others” and thus increasing empathy for those living with HIV [18]. It is also known that increasing knowledge about HIV can also increase HIV testing and reduce HIV stigma [21, 22].

To date, only three randomized clustered trials have examined effects on HIV stigma in African American churches. A pilot cluster randomized clinical trial (RCT) conducted in Kansas City with African American churches found increases in testing [23] but no reduction in HIV stigma [24]. Another pilot cluster RCT conducted with African American and Latino churches in Los Angeles also found increases in testing across both types of churches and reduction in HIV stigma among Latino churches but not African American churches [25]. However, a third pilot cluster RCT found reduced stigma among participants in African American churches in the rural South [26]. These mixed findings suggest there’s still much to learn about how to reduce stigma in African American churches.

Taking It to the Pews (TIPS) is a full-scale cluster RCT using a community-based participatory research approach and full partnership with faith leaders. The design and implementation of TIPS used a framework guided by an ecologically expanded version of the Theory of Planned Behavior (TPB), which was a combination of TPB and the Social-Ecological model [27]. The focus of the project was to test a multilevel, religiously tailored intervention against a standard, multilevel HIV information intervention on HIV testing rates among African American adult church members and community members (results reported elsewhere). The purpose of this paper is to (a) examine the TIPS intervention impact on HIV-related stigma outcomes as compared to the standard HIV information intervention and (b) conduct a process evaluation to understand the relationship between exposure to the multilevel TIPS intervention components and HIV knowledge along with HIV-related stigma.

## Methods

### Study Design and Participants

The study design and procedures are fully described in a published protocol paper [27]. Briefly, the study was a 2-arm, parallel-group, cluster-designed RCT with 14 participating churches and assessments at baseline, 6 months, and 1-year follow-up with recruited participants from each church. Additionally, exposure to TIPS intervention arm components was examined using participants’ self-reports on the 12-month survey. The participating African American churches (i.e., churches that primarily serve African American congregants and other community members) were recruited from the Kansas City metropolitan area through group informational meetings and individual church follow-up with the assistance of Calvary Community Outreach Network (CCON), a faith-based community partner that was engaged in all aspects of the study. Church eligibility criteria included having (a) ≥ 150 adult church members in attendance at Sunday services; (b) a church outreach ministry (e.g., food/clothing pantry, social services, daycare) that served ≥ 50 adult community members (annually) who received services at least 4 times/year; (c) the pastor’s commitment to deliver assigned study activities; (d) two or three church members who agreed to serve as church health liaisons (CHLs), designated by the pastor to coordinate and deliver all study activities assigned to their respective arm; and (e) pastor and CHLs’ commitment to receive initial and ongoing study training.

Study team members recruited community members at church events (e.g., Sunday services, mid-week Bible studies, and outreach ministries such as food and clothing pantries) using a self-report screening survey. Eligibility criteria included (a) aged 18 to 64; (b) willing to participate in surveys after church services (baseline, 6-month, and 12-month); (c) willing to provide contact information (i.e., two phone numbers, mailing/email address, phone numbers for two persons with whom they have ongoing contact; and (d) regularly attended church (at least once a month) or used church outreach services (at least four times per year). The University of Missouri–Kansas City Institutional Review Board approved this study, and participants provided written informed consent.

To facilitate implementation, the study was implemented across two waves of churches sequentially (*n* = 6 in wave one and *n* = 8 in wave two). Within each cohort, churches were stratified by size and denomination and randomly assigned to the TIPS intervention or the comparison arms by the statistician (last author) using a numbered list of participating churches based on a computer-based random number-generated allocation scheme [27]. Participants were recruited within their participating churches and received study activities per their churches’ assigned arm. The nature of the intervention precluded blinding of participating churches, study staff, or participants.

### Intervention Description

As detailed elsewhere, this trial compared a multilevel, religiously tailored intervention to a multilevel, non-tailored, standard education condition [27]. The research team trained pastors and CHLs to deliver one to two culturally and religiously tailored materials/activities each month from the TIPS HIV Tool Kit through multilevel church outlets including (a) individual/person-to-person contacts (e.g., print materials, phone/email/text messages); (b) ministry groups (e.g., HIV educational games, printed/video testimonials, facilitator discussion guides); (c) church services (e.g., sermons, responsive readings, church bulletins, pastor-modeled receipt of HIV testing); and (d) community outreach ministries (e.g., brochures, resource tables, posters). Comparison churches’ CHLs were trained to deliver non-tailored HIV testing event announcements, brochures, and resource information from the local health department and other public health sources through their multilevel outlets. CHLs in both study arms were trained to coordinate their churches’ HIV testing events with the Kansas City, MO Health Department, the study’s health partner. All churches were charged with coordinating two HIV testing events—one during/after church services and one during outreach activities.

### Measures

Baseline, 6-month, and 12-month surveys assessed participant characteristics, exposure to intervention activities, and outcomes. The primary outcomes for the current study (HIV-related stigma and HIV knowledge) were assessed with self-reported measures. Participants also indicated their exposure to 14 individual study activities (e.g., church bulletins, announcements, sermons about HIV, responsive readings, and text messages).

#### Dependent Variables

We adapted items from prior studies that measured the following aspects of HIV stigma:

1. *HIV stigma—discomfort* (instrumental aspect of stigma): five items that asked about how comfortable respondents would feel being around people with HIV in various community settings (school, restaurant, grocery store, and two questions about church interactions) [24, 28], using responses on a 5-point Likert scale from very comfortable to very uncomfortable (*α* = 0.87 in our sample).
2. *HIV stigma—anticipated stigma or rejection* (symbolic aspect of stigma): four items about whether respondents endorsed beliefs that if they had HIV, they would be rejected, fired, couldn’t face their families, or might be treated differently or discriminated against [29, 30], using responses on a 5-point Likert scale from strongly disagree to strongly agree (*α* = 0.77 in our sample).
3. *HIV stigma—overall stigma*: a total of nine items that included both the discomfort and anticipated stigma scales, as well as two items adapted from Herek and Capitano [31]: one item about the extent to which respondents endorsed the belief that people with HIV are “responsible” for their illness (5-point Likert scale from strongly disagree to strongly agree) and another item that asks individuals about how “afraid” they are of people with HIV (5-point Likert scale from not at all afraid to very afraid) (*α* = 0.79 for the 11-item scale in our sample).
4. *HIV knowledge*, 16 items adapted from the HIV Knowledge Questionnaire (HIV-KQ- 18)[32]. These items included questions such as “A person can get HIV by using a cup or plate that has been used by a person with HIV/AIDS and “HIV can be transmitted through mosquito bites,” with “true, false, or don’t know” as responses; items were scored “1” for correct answers and “0” for incorrect or “don’t know” answers (*α* = 0.96 in our sample). HIV knowledge was included as a control variable in the regressions examining the stigma outcomes, given that it is known to be a component of stigma [24].

#### Control Variables

Respondents provided information on various types of demographic and socio-economic backgrounds. We included the following control variables, given their association with HIV stigma in previous church-based studies [22, 33]: *age* (18–29 (reference group), 30–49, and 50–64); *gender* was defined as male (reference group) and female; and *highest level of education completed*, with categories of high school or less (reference group), some college/associates degree, or 4-year college degree or more. In addition, we included *sexual orientation or identity*, with categories of heterosexual (reference group), lesbian, gay, or bisexual, other, refused or missing); *marital status*, with categories of never married (reference group), married/partnered, or divorced/separated/widowed; *ever tested for HIV* (yes/no) and *ever tested for sexually transmitted infections or STI* (chlamydia, gonorrhea, or syphilis; yes/no); *any sexual risk in the last 12 months* (report of 4 or more sexual partners, no condom use, a diagnosis of an STI, injection drug use or having exchanged sex for drugs or money); and *any HIV risk in the last 12 months* (any report of injection drug use, shared needles, drug retreatment, homeless, in a correctional facility, victim of domestic violence, exchange of sex for drugs, sex with a former prisoner, an injection drug user or a man who has sex with men).

We also controlled for whether or not a respondent knew anyone (friends, family, co-workers, others) who “has HIV or AIDS or has died of HIV” (yes/no), which has been shown to be related to HIV stigma [34–36]. For *drug addiction stigma and homosexuality stigma*, we used an adapted 5-item scale on the extent to which respondents endorsed attitudes towards alcoholics [37], changing “alcoholics” and “alcoholism” to “drug addicts” and “drug addiction”[38] (*α* = 0.90 in our sample). For homosexuality stigma, we used 6 items from the 19-item Heterosexual Attitudes Towards Homosexuality (HATH) scale [39] and added one item (“Homosexuals should be barred from the clergy”). For all variables, response categories ranged from “disagree strongly” to “agree strongly” (*α* = 0.82 in our sample). Both the adapted drug addiction and homosexuality stigma scales were used in previous church-based studies [33, 40].

Finally, because of the two sampling approaches at the study churches—church members vs. community members who participate in the study church’s service programs—we included an identifier for each of these types of study participants (church member vs. outreach ministry service program participant). In addition, we included an indicator for wave to control for any possible wave/time differences.

### Analysis

Analyses of intervention effects on HIV knowledge and HIV-related stigma from baseline to 6 and 12 months were performed using multilevel, multivariable mixed models with fixed-effect terms for the experimental condition and random-effect terms for churches, the unit of randomization. A secondary analysis estimated associations between study outcomes (HIV discomfort and anticipated stigma, overall HIV stigma, and HIV knowledge) and type and number of intervention activities participants reported by intervention group participants exposed during the 12-month intervention. Stata statistical software version 17.0 (StataCorp LLC, College Station, TX) was used for data analyses to determine if there was a difference in HIV knowledge and HIV-related stigma (HIV discomfort and anticipated HIV stigma and overall HIV stigma) over time between TIPS intervention and standard information groups after adjusting for covariates. We also explored unadjusted changes by arm in HIV knowledge and stigma scores over time (baseline, 6 months, 12 months, and 6 and 12 months combined) using the mixed models and clustering by churches to examine trends. Overall follow-up rates for church and community member participants were 49% at 6 months and 47% at 12 months, although 62% of the sample is included in our analyses since people contributed if they completed the 6- or 12-month time points. Given this, we also examined differences in participant characteristics among those who were lost to follow-up (baseline only) and those who completed at least one follow-up.

## Results

A total of 1491 persons were enrolled (780 across 7 intervention churches and 711 across 7 comparison churches; see Table 1). Church members (as compared to community members receiving outreach services at the church) represented 57% of participants in intervention churches and 81% in comparison churches (*p* < 0.001). Other statistically significant differences between intervention and comparison church participants included age (*p* = 0.006), education (*p* = 0.003), sexual orientation/identification (more non-heterosexual people participating in intervention churches, *p* = 0.038), and anticipated HIV stigma (higher among intervention church participants, *p* = 0.002). Fifty-four percent of intervention participants and 67% of comparison participants completed at least one follow-up survey. Participants lost to follow-up were different from those who completed at least one follow-up in several ways, as shown in the Supplemental Table. For example, those lost to follow-up were more likely to be church members, of older age, female, of higher education, married/partnered, know someone with HIV, have lower overall stigma and HIV discomfort, higher homosexuality stigma and lower drug stigma, and lower sexual and HIV risk.

**Table 1:** Characteristics of Taking It to the Pews participants (*n* = 1491)

Participant characteristics	Total	Comparison	Intervention	*p*-value
	*N* = 1491	*N* = 711	*N* = 780	
Church member				
No	476 (32%)	137 (19%)	339 (43%)	**< 0.001**
Yes	1015 (68%)	574 (81%)	441 (57%)	
Age (years), mean (SD)	43.6 (12.7)	43.8 (13.1)	43.3 (12.4)	0.48
Age group (years)				
18–29	262 (18%)	132 (19%)	130 (17%)	**0.006**
30–49	630 (42%)	270 (38%)	360 (46%)	
50–64	599 (40%)	309 (43%)	290 (37%)	
Sex				
Male	473 (32%)	238 (34%)	235 (30%)	0.17
Female	1012 (68%)	471 (66%)	541 (70%)	
Education				
HS or less	517 (35%)	219 (32%)	298 (39%)	**0.003**
Some college/associates	614 (42%)	323 (46%)	291 (38%)	
4Y degree +	327 (22%)	153 (22%)	174 (23%)	
Sexual identification				
Heterosexual	1404 (95%)	678 (97%)	726 (94%)	**0.038**
Lesbian, gay, bisexual	27 (2%)	11 (2%)	16 (2%)	
Other	44 (3%)	13 (2%)	31 (4%)	
Marital status				
Never married	610 (41%)	292 (41%)	318 (41%)	0.58
Married/partnered	542 (37%)	252 (36%)	290 (38%)	
Div/sep/widow	323 (22%)	162 (23%)	161 (21%)	
Know someone with HIV				
No	844 (58%)	399 (58%)	445 (59%)	0.60
Yes	604 (42%)	294 (42%)	310 (41%)	
Overall stigma (11 items)	27.6 (7.5)	27.2 (7.6)	28.0 (7.4)	**0.029**
Discomfort scale	11.1 (5.0)	11.0 (5.1)	11.2 (5.0)	0.36
Anticipated stigma scale	11.4 (3.7)	11.1 (3.7)	11.7 (3.7)	**0.002**
Attitudes towards homosexuality score	3.0 (0.9)	3.0 (0.9)	3.0 (0.9)	0.32
Drug stigma	2.5 (1.0)	2.5 (1.0)	2.4 (1.0)	0.30
Ever tested for HIV				
No	309 (22%)	152 (23%)	157 (22%)	0.59
Yes	1,068 (78%)	507 (77%)	561 (78%)	
Ever STI testing* at baseline				
No	405 (30%)	196 (30%)	209 (29%)	0.78
Yes	961 (70%)	457 (70%)	504 (71%)	
Any sexual risk: last 12 M				
No	1229 (82%)	590 (83%)	639 (82%)	0.59
Yes	262 (18%)	121 (17%)	141 (18%)	
Any HIV risk: last 12 M				
No	996 (67%)	476 (67%)	520 (67%)	0.91
Yes	495 (33%)	235 (33%)	260 (33%)	
HIV knowledge score	11.0 (2.9)	11.0 (2.9)	11.0 (2.9)	0.90

^*^Tested for gonorrhea, chlamydia, and/or syphilis

Significant differences (*p*<.05) between Comparison and Intervention participants are bolded

Table 2 provides the independent associations between the intervention and the four outcomes (HIV discomfort, anticipated HIV stigma, overall HIV stigma, and HIV knowledge) while controlling for covariates. Overall, the TIPS intervention was associated with a higher anticipated HIV stigma (*β* = 0.44 (95% confidence interval (CI) 0.06, 0.82)) as compared to the standard information comparison condition. The intervention was not associated with any changes in the HIV discomfort, overall HIV stigma, or HIV knowledge scores. In terms of covariates, being a church member (vs. a community member receiving church outreach services) was associated with lower HIV stigma scores (discomfort, anticipated, and overall stigma scores). Knowing someone with HIV was consistently associated with lower HIV stigma scores and higher HIV knowledge. Greater drug stigma was associated with higher HIV stigma scores and lower HIV knowledge; greater homosexuality stigma was associated with higher discomfort and anticipated stigma scores. As expected, greater HIV knowledge was associated with less HIV discomfort and anticipated stigma.

**Table 2:** Associations with stigma and HIV knowledge among Taking It to the Pews participants

	Overall stigma score	Discomfort score	Anticipated stigma score	HIV knowledge score
Intervention	0.54 (− 0.40, 1.47)	0.07 (− 0.60, 0.75)	**0.44 (0.06, 0.82)**	0.11 (− 0.20, 0.41)
Church member	** − 1.82 (− 2.63, − 1.01)**	** − 1.03 (− 1.59, − 0.47)**	** − 0.66 (− 1.08, − 0.23)**	− 0.13 (− 0.45, 0.18)
Study wave 2	− 0.57 (− 1.50, 0.36)	− 0.34 (− 1.01, 0.33)	− 0.16 (− 0.53, 0.22)	0.00 (− 0.30, 0.30)
Age (years)	0.00 (− 0.03, 0.03)	− 0.01 (− 0.03, 0.01)	0.01 (− 0.00, 0.03)	− 0.01 (− 0.02, 0.01)
Female	− 0.01 (− 0.80, 0.78)	− 0.25 (− 0.80, 0.30)	0.40 (− 0.02, 0.81)	0.16 (− 0.14, 0.47)
Education				
HS or less	**–**	**–**	**–**	**–**
Some college or associates	− 0.69 (− 1.53, 0.15)	− 0.51 (− 1.09, 0.07)	− 0.10 (− 0.54, 0.34)	**0.97 (0.64, 1.29)**
4-year degree or more	− 0.90 (− 1.91, 0.12)	** − 1.08 (− 1.79, − 0.37)**	0.09 (− 0.45, 0.63)	**1.61 (1.22, 2.00)**
Sexual identification				
Heterosexual	**–**	**–**	–	**–**
Lesbian, gay, or bisexual	1.91 (− 0.82, 4.65)	1.18 (− 0.72, 3.07)	1.18 (− 0.27, 2.63)	0.60 (− 0.47, 1.66)
Other	0.22 (− 1.65, 2.09)	0.40 (− 0.90, 1.70)	− 0.32 (− 1.31, 0.68)	** − 1.33 (− 2.05, − 0.60)**
Marital status				
Never married	–	–	–	**–**
Married/partnered	− 0.73 (− 1.55, 0.10)	− 0.20 (− 0.77, 0.37)	** − 0.46 (− 0.89, − 0.02)**	0.24 (− 0.08, 0.56)
Divorced, widowed, or separated	− 0.62 (− 1.60, 0.36)	0.10 (− 0.58, 0.78)	** − 0.58 (− 1.10, − 0.06)**	0.19 (− 0.19, 0.57)
Ever tested for HIV	− 0.03 (− 0.86, 0.80)	− 0.15 (− 0.73, 0.42)	0.03 (− 0.41, 0.47)	**1.22 (0.90, 1.53)**
Ever tested for STI	** − 0.90 (− 1.67, − 0.12)**	** − 0.67 (− 1.21, − 0.13)**	− 0.33 (− 0.74, 0.08)	**0.67 (0.37, 0.97)**
Sexual risk last 12 M	**1.03 (0.08, 1.98)**	0.29 (− 0.37, 0.95)	**0.78 (0.27, 1.28)**	** − 0.41 (− 0.78, − 0.04)**
HIV risk last 12 M	0.57 (− 0.20, 1.34)	0.26 (− 0.27, 0.79)	0.32 (− 0.09, 0.72)	0.00 (− 0.30, 0.30)
Know someone w/HIV	** − 1.89 (− 2.61, − 1.18)**	** − 1.12 (− 1.62, − 0.63)**	** − 0.39 (− 0.77, − 0.01)**	**0.53 (0.26, 0.81)**
HIV knowledge score	** − 0.54 (− 0.67, − 0.40)**	** − 0.38 (− 0.47, − 0.28)**	** − 0.11 (− 0.19, − 0.04)**	–
Attitudes Towards Homosexuality score	**1.32 (0.89, 1.76)**	**0.36 (0.05, 0.66)**	**0.60 (0.37, 0.83)**	− 0.12 (− 0.29, 0.05)
Drug stigma score	**1.35 (0.97, 1.74)**	**0.64 (0.37, 0.91)**	**0.38 (0.18, 0.59)**	** − 0.43 (− 0.58, − 0.28)**

Significant associations (*p*<.05) are bolded

Table 3 provides process evaluation data related to dose response for intervention church participants. There was a negative relationship between the total number of exposures to intervention activities and the HIV stigma outcomes (more exposures, lower stigma) and a positive relationship between exposures and HIV knowledge (more exposures, higher knowledge). Across different types of intervention activities, all associations were in the expected directions (more exposures and lower stigma, more exposures, and higher HIV knowledge). Of note, exposures to different intervention activities were most consistently associated with improved HIV knowledge (9 of the 15 types of activities) and reduced HIV discomfort and overall HIV stigma (6 of the 15 types of activities for each stigma measure). Only two types of activities were associated with reduced anticipated HIV stigma: having attended an HIV testing event and having health professionals or HIV + people provide information about HIV; these same activities were also associated with lower discomfort and overall HIV stigma and higher HIV knowledge and in fact were the only activities that were associated with all four outcomes.

**Table 3:** Associations between exposures and overall, discomfort and anticipated stigma, and HIV knowledge among intervention participants in Taking It to the Pews

	Overall stigma	Discomfort	Anticipated	HIV knowledge
Number of study exposures reported	** − 0.18 (− 0.31, − 0.053)**	** − 0.10 (− 0.18, − 0.03)**	** − 0.06 (− 0.12, − 0.00)**	**0.08 (0.03, 0.12)**
Printed materials	** − 1.35 (− 2.56, − 0.14)**	** − 0.86 (− 1.61, − 0.10)**	− 0.52 (− 1.09, 0.05)	**0.85 (0.42, 1.28)**
Heard the pastor deliver sermon	** − 1.41 (− 2.60, − 0.22)**	− 0.73 (− 1.48, 0.01)	− 0.51 (− 1.07, 0.05)	**0.92 (0.50, 1.34)**
Participated in responsive reading	− 0.87 (− 2.07, 0.33)	− 0.06 (− 0.81, 0.69)	− 0.56 (− 1.12, 0.01)	0.32 (− 0.11, 0.76)
See HIV/AIDS poster or bulletin board	− 0.80 (− 1.97, 0.37)	− 0.22 (− 0.96, 0.51)	− 0.52 (− 1.08, 0.05)	**0.47 (0.05, 0.89)**
Seen HIV/AIDS resource table	− 0.87 (− 2.04, 0.30)	− 0.36 (− 1.09, 0.37)	− 0.56 (− 1.11, 0.00)	**0.53 (0.11, 0.95)**
Watched video testimony	0.69 (− 0.55, 1.94)	0.60 (− 0.18, 1.38)	0.10 (− 0.50, 0.70)	− 0.02 (− 0.47, 0.43)
Received printed story or testimonial	− 0.68 (− 1.87, 0.52)	− 0.28 (− 1.03, 0.47)	− 0.25 (− 0.82, 0.32)	**0.66 (0.23, 1.08)**
Attended HIV testing event	** − 1.72 (− 2.91, − 0.52)**	** − 0.90 (− 1.65, − 0.15)**	** − 0.69 (− 1.26, − 0.12)**	**0.58 (0.15, 1.01)**
Participated in HIV educational game	− 0.16 (− 1.75, 1.43)	− 0.46 (− 1.46, 0.54)	0.32 (− 0.39, 1.02)	− 0.01 (− 0.58, 0.56)
Received wallet-sized information card	− 0.63 (− 1.98, 0.73)	− 0.52 (− 1.37, 0.32)	0.14 (− 0.52, 0.80)	0.2 (− 0.22, 0.76)
Health professional or HIV + person provided information	** − 1.72 (− 2.86, − 0.58)**	** − 1.01 (− 1.72, − 0.30)**	** − 0.57 (− 1.12, − 0.02)**	**0.63 (0.22, 1.04)**
Received phone calls or text messages	− 0.81 (− 2.00, 0.38)	− 0.86 (− 1.60, − 0.12)	− 0.02 (− 0.59, 0.55)	0.41 (− 0.02, 0.84)
Received emails	− 1.02 (− 2.22, 0.18)	− 0.50 (− 1.25, 0.24)	− 0.45 (− 1.04, 0.13)	0.10 (− 0.33, 0.54)
Pastor or church leader tested	** − 1.51 (− 2.71, − 0.30)**	** − 1.09 (− 1.84, − 0.34)**	− 0.37 (− 0.94, 0.20)	**0.98 (0.54, 1.41)**
Church website or Facebook	** − 1.32 (− 2.55, − 0.09)**	** − 0.84 (− 1.61, − 0.08)**	− 0.53 (− 1.13, 0.06)	**0.45 (0.00, 0.89)**

Significant associations (*p*<.05) are bolded

An examination of overall differences in the primary outcomes between intervention and comparison arms over time found that both arms had modest increases in mean HIV knowledge (11.0 to 11.6 intervention and 10.8 to 11.4 comparison) and modest decreases in discomfort (11.2 to 10.1 intervention and 10.8 to 10.1 comparison), anticipated stigma (11.7 to 11.3 intervention and 10.9 to 10.8 comparison), and overall stigma score (28.0 to 26.1 intervention and 26.7 to 25.7 comparison), respectively.

## Discussion

In this cluster RCT of a faith-based, multilevel intervention with 14 African American churches in Kansas City, MO, we found no overall reductions in HIV-related stigma or improvements in HIV knowledge when comparing TIPS intervention and comparison churches. In fact, the TIPS intervention was associated with *higher* anticipated HIV stigma at follow-up. These findings suggest stigma dynamics within faith-based settings that require nuanced interpretation. Importantly, a process evaluation found that greater exposure to intervention activities was associated with reduced stigma and greater knowledge among participants, both in terms of dose response (more exposures, lower stigma, and greater knowledge) and with particular types of activities. The unexpected increase in anticipated HIV stigma among intervention participants warrants careful interpretation. The anticipated stigma measure did not ask specifically about anticipated stigma at church, which is where one might expect most immediate effects of a church- and faith-based intervention. In addition, it is important to emphasize that the intervention’s design, which primarily focused on HIV testing, may have been insufficient to comprehensively address the complex nature of HIV-related stigma. We elaborate on these findings and their implications below.

Although TIPS was not designed primarily to reduce stigma, this full-scale efficacy trial provided a practical opportunity to learn about TIPS’ possible impact on HIV stigma, which is important because to date only two prior RCTs of faith-based interventions have been published that had stigma reduction as the primary outcome and both had mixed results [25, 26]. One study was a pilot cluster RCT that found HIV stigma reductions in the Latino intervention church as compared to its matched control church, yet did not find reductions in the African American intervention church compared to its matched control church, though statistically significant increases in HIV testing were found across Latino and African American churches [25]. The other study included only African American churches and did find statistically significant reductions in HIV stigma but (a) only measured stigma among people who participated in an 8-h curriculum and (b) only for “individual-level (personally held) HIV stigma” and not in the other measure of stigma that was individuals’ perceptions of others in their congregation or community (“community-level stigma”) [26]. The stigma findings of the TIPS trial along with these two other studies suggest that changing HIV stigma beliefs is more difficult than increasing uptake of HIV testing. Previous research on stigma reduction interventions more generally have found that *informational* and *contact* components are both necessary to increase empathy for those who are stigmatized and in so doing, reduce stigma [41–43]. TIPS was comprehensive on informational components about HIV but less so on tools to increase contact with people with HIV, suggesting areas for future modification of the TIPS toolkit.

Another aspect of the TIPS cluster RCT to consider in the context of the results on stigma is that comparison churches’ CHLs implemented an HIV intervention of similar intensity by delivering non-tailored HIV testing event announcements, brochures, and resource information from their local health department and other public health resources through their multilevel outlets. In addition, CHLs in both study arms were trained to coordinate their churches’ two HIV testing events with the Kansas City, MO Health Department. Although the comparison churches’ intervention materials were not religiously tailored, they were delivered by faith leaders, which introduces some degree of religious tailoring. This could be why improvements in HIV knowledge and stigma were found in both arms of the study, which might have been different had the comparison arm been a true control arm. Also, the overall analyses assessed HIV stigma and knowledge change among study participants regardless of their exposure to the intervention.

The reason that TIPS intervention churches had higher anticipated stigma at follow-up as compared to comparison churches is not clear. As noted earlier, previous faith-based interventions have found reductions in individual-level (personally held) HIV stigma but not in their perceptions of stigma among others in their congregation or community [26]. In addition, analyses of the TIPS baseline data found that knowing someone with HIV, which is often associated with lower HIV stigma [28, 33–36], was negatively associated with HIV discomfort, but was not associated with anticipated stigma, suggesting that individuals may perceive that community attitudes are harder to change than their own attitudes [40]. Of note, the anticipated stigma measure used items from previous research [29, 30], but none of these were specifically focused on anticipated stigma within the church community (instead on concerns if they had HIV about being “rejected” or “treated differently or discriminated against” in general and specifically within workplaces (“fired”) and not being able to face their families). HIV stigma measures need further refinement to distinguish between sources and settings of HIV stigma (e.g., church, family, African American community, workplace, health organizations) to be able to inform more targeted HIV intervention strategies.

The statistically significant relationships between key covariates and the HIV stigma and knowledge outcomes are informative for interpreting the current study’s findings as well as for interventions that aim to reduce HIV stigma in similar populations. Knowing someone with HIV was negatively associated with all stigma outcomes and positively associated with HIV knowledge, confirming social psychological theories of stigma reduction, including the contact hypothesis, which posits that direct or indirect contact can engender empathy for people with a stigmatized condition [44]. In addition, the negative association between HIV knowledge and all our stigma measures is consistent with the literature, and, together, these findings demonstrate the importance of both informational and contact components for reducing stigma [41–43]. Homosexuality and drug addiction stigma scores were also positively associated with all HIV stigma measures, which is consistent with the few church-based studies that have assessed these other types of stigmas with HIV stigma [25, 40]. In terms of HIV knowledge, drug addiction stigma was negatively associated, although the homosexuality stigma score was not statistically significant, suggesting that changing attitudes around homosexuality requires more than improving knowledge about HIV. Although attitudes towards homosexuality are changing in society at large and in some churches, traditional doctrines that condemn same sex relations make this topic taboo in many Bible-based, fundamentalist churches [45, 46].

The analyses of dose response among TIPS intervention participants provide some additional insights into the design of faith-based HIV interventions that are particularly promising in terms of reducing HIV stigma. Greater exposure to more types of TIPS activities was associated with lower HIV stigma and greater HIV knowledge, which is consistent with previous church-based interventions that examined results in relation to exposures, including studies of HIV stigma [47]. Interestingly, two specific types of exposures were consistently related to all the HIV stigma and knowledge outcomes: having attended an HIV testing event and receiving information from a health professional or HIV-positive person were each associated with lower HIV stigma (overall, discomfort, and anticipated) and greater HIV knowledge. These findings confirm the importance partnering with local health departments or HIV service organizations and involving people with lived HIV experience in intervention activities to reduce HIV stigma. Previous research has found the importance of external partnerships for congregation-based health programming in general [48–50] and for HIV in particular [51–54] and can contribute to the sustainability of congregation-based HIV efforts [18].

Further, our findings demonstrate that HIV testing and stigma reduction are mutually facilitative in congregational settings [18] and that involving health professionals in congregational HIV activities may help “normalize” HIV as a health issue rather than a moral issue [19, 20]. Seeing the pastor or a church leader get tested and seeing HIV information on the church website or Facebook page were also associated with lower overall HIV stigma and discomfort and higher HIV knowledge among participants, which further demonstrates the importance of pastor and institutional support for HIV programs [55, 56]. Nevertheless, these were not sufficient for reducing anticipated stigma, suggesting that other intervention tools and strategies specific to HIV stigma may be needed in not only in churches and but also in other places (e.g., home, work, healthcare, recreational settings) for people to feel that they would not be rejected or discriminated against if they had HIV—and to possibly remove existing stigmatizing actions that may be perpetuated in these settings.

The current study had several limitations. First, overall follow-up rates were just under 50%, with 62% of the sample having completed at least one follow-up; although not uncommon for community-based longitudinal studies, this could have affected our findings in any number of directions. For example, the characteristics of participants lost to follow-up were different from those with at least one follow-up in many ways. The low follow-up rate and participant imbalances between intervention and comparison groups potentially introduce selection bias and reduce the study’s representativeness. Second, the study was conducted with African American churches in Kansas City, which may further limit generalizability of findings. Third, all our outcomes were self-reported and could have been affected by social desirability and/or measurement biases. Fourth, more dimensions of HIV stigma may need to be assessed to better understand drivers of stigma and thereby improve the ability to enhance the design of HIV interventions to not only shift HIV testing behaviors but also stigmatizing beliefs. The stigma measurement approach in the TIPS study may not have captured the full complexity of HIV-related stigma, particularly its intersectional and contextual nuances. Fifth, the design of the intervention was primarily to increase uptake of HIV testing with few TIPS tools designed specifically to address HIV stigma, which may have inadvertently constrained its effectiveness in comprehensively addressing HIV-related stigma. Finally, there was a larger proportion of community members (43%) in intervention churches, whereas they represented only 19% of participants in comparison churches. The intervention was designed primarily to be implemented through church channels (e.g., worship services, Bible studies, ministry groups) that reached church members more frequently with some limited and more passive and less frequent activities (brochures, resource tables, posters) disseminated through church outreach ministries with community members. This means that a lower proportion of participants in the intervention churches were exposed through church channel activities than if there had been a similar distribution of church members in both arms.

Nevertheless, the current study makes several contributions to the literature on faith-based HIV stigma reduction programs. For example, as recommended by Sengupta et al. [57], the TIPS study used multi-dimensional stigma instruments that underwent psychometric analysis and an RCT study design. Further, as a church-level study, we measured changes across all participants regardless of participation in the various intervention activities but also conducted a process evaluation and secondary analysis to understand dose–response and exposure related to outcomes. Further, it should be noted that, to our knowledge, no fully powered faith-based HIV multi-level HIV intervention trials have been designed specifically to address HIV stigma nor have been reported in the literature. Even with the limitations of TIPS tools being tailored for HIV testing and not stigma, our findings can inform future iterations of the TIPS toolkit and other faith-based HIV interventions. Specifically, TIPS components such as contact with health professionals (HIV education) and people living with HIV (testimonials) along with congregation-wide HIV testing events and pastors modeling receipt of testing while addressing stigma are promising components for faith-based interventions that also aim to reduce stigma. Additional tools and strategies may be needed for stigma reduction more generally in the congregation and community, but HIV stigma reduction and HIV testing appear to have synergistic effects in faith-based settings.

## Supplementary Information

Below is the link to the electronic supplementary material.

Supplementary file1 (PDF 203 KB)

## Data Availability

De-identified data from this study will be made available (as allowable according to institutional IRB standards) by emailing the corresponding author and completing a registration process.

## Code Availability

Relevant analysis code will be made available by emailing the corresponding author.
