# Supplementary material for: Effects of a Faith-Based, Multilevel Intervention on HIV-Related Stigma and HIV Knowledge Among African American Church-Affiliated Populations
Source: J Racial Ethn Health Disparities. 2025 Apr 25;13(4):2523–34. doi: 10.1007/s40615-025-02436-3 (PMC12903788; doi:10.1007/s40615-025-02436-3)
Supplement: Supplementary file 1 — Supplementary file1 (PDF 203 KB) [file 40615_2025_2436_MOESM1_ESM.pdf]

Supplementary Information

Supplemental Table. Participant Characteristics by Completion of At Least One Follow-Up Visit, Taking It to the Pews

Participant Characteristics	Total N=1,491	Baseline Only N=568	Follow-up N=923	p-value
Church Member				
No	476 (32%)	130 (23%)	346 (37%)	<0.001
Yes	1,015 (68%)	438 (77%)	577 (63%)	
Age (years), mean (SD)	43.6 (12.7)	45.7 (11.7)	42.2 (13.2)	<0.001
Age group (years)				
18-29	262 (18%)	66 (12%)	196 (21%)	<0.001
30-49	630 (42%)	245 (43%)	385 (42%)	
50-64	599 (40%)	257 (45%)	342 (37%)	
Sex				
Male	473 (32%)	145 (26%)	328 (36%)	<0.001
Female	1,012 (68%)	420 (74%)	592 (64%)	
Education				
HS or less	517 (35%)	151 (27%)	366 (41%)	<0.001
Some college/Associates	614 (42%)	249 (45%)	365 (40%)	
4Y Degree+	327 (22%)	156 (28%)	171 (19%)	
Sexual identification				
Heterosexual	1,404 (95%)	538 (96%)	866 (95%)	0.26
Lesbian, gay, bisexual	27 (2%)	12 (2%)	15 (2%)	
Other	44 (3%)	12 (2%)	32 (4%)	
Marital status				
Never married	610 (41%)	202 (36%)	408 (45%)	0.004
Married/Partnered	542 (37%)	228 (41%)	314 (34%)	
Div/Sep/Widow	323 (22%)	132 (23%)	191 (21%)	
Know someone with HIV				
No	844 (58%)	300 (54%)	544 (61%)	0.010
Yes	604 (42%)	255 (46%)	349 (39%)	
Overall Stigma (11 items)	27.6 (7.5)	26.6 (7.4)	28.2 (7.5)	<0.001
Discomfort Scale	11.1 (5.0)	10.5 (4.9)	11.5 (5.0)	<0.001
Anticipated Stigma Scale	11.4 (3.7)	11.2 (3.5)	11.5 (3.8)	0.094
Attitudes towards homosexuality score	3.0 (0.9)	3.1 (0.8)	2.9 (0.9)	<0.001
Drug stigma	2.5 (1.0)	2.4 (0.9)	2.5 (1.0)	0.036
Ever tested for HIV				
No	309 (22%)	124 (23%)	185 (22%)	0.46
Yes	1,068 (78%)	404 (77%)	664 (78%)	
Ever STI testing at Baseline				
No	405 (30%)	154 (29%)	251 (30%)	0.90

Participant Characteristics	Total N=1,491	Baseline Only N=568	Follow-up N=923	p-value
Yes	961 (70%)	369 (71%)	592 (70%)	
Any sexual risk: last 12M				
No	1,229 (82%)	499 (88%)	730 (79%)	<0.001
Yes	262 (18%)	69 (12%)	193 (21%)	
Any HIV risk: last 12M				
No	996 (67%)	415 (73%)	581 (63%)	<0.001
Yes	495 (33%)	153 (27%)	342 (37%)	
HIV Knowledge Score	11 (3)	11 (3)	11 (3)	0.013
